# Axial length, myopia progression, and myopic maculopathy in Stickler syndrome

**DOI:** 10.1111/aos.70030

**Published:** 2025-11-18

**Authors:** Kirstine B. Boysen, Mette Bertelsen, Nanna D. Rendtorff, Stense Farholt, Line Kessel

**Affiliations:** ^1^ Department of Ophthalmology Copenhagen University Hospital – Rigshospitalet Copenhagen Denmark; ^2^ Department of Clinical Medicine University of Copenhagen Copenhagen Denmark; ^3^ Department of Clinical Genetics Copenhagen University Hospital – Rigshospitalet Copenhagen Denmark; ^4^ Center for Rare Disease, Department of Pediatrics and Adolescent Medicine Copenhagen University Hospital – Rigshospitalet Copenhagen Denmark; ^5^ Center for Rare Disease, Department of Pediatrics and Adolescent Medicine Aarhus University Hospital Aarhus Denmark

**Keywords:** *COL11A1*, *COL2A1*, degenerative myopia, myopia, myopic maculopathy, type 1 Stickler syndrome, type 2 Stickler syndrome

## Abstract

**Purpose:**

We lack knowledge on the potentially progressive nature of and the prevalence of complications to myopia as a characteristic trait of Stickler syndrome.

**Methods:**

This cross‐sectional study combines ophthalmic examination and medical record data on Danish patients with genetically confirmed Stickler syndrome type 1 (*COL2A1*) and type 2 (*COL11A1*). The main outcomes are axial length, spherical equivalent refraction (SER), SER over time, and myopic maculopathy category by fundus photography.

**Results:**

The study includes 71 patients with type 1 (age: median = 29 years, IQR = 15–49 years; 44% male) and 13 with type 2 Stickler syndrome (age: median = 27 years, IQR = 9–33 years; 69% male). For type 1, the median SER was −6.00 dioptres (D) (IQR = −8.88 to −2.19 D) and −6.75. (IQR = −10.88 to −1.94) for type 2, (*p* = 0.52). Mean axial length was 25.99 ± 1.99 and 26.55 ± 3.45 mm, respectively (*p* = 0.57). SER was nonprogressive in childhood in both subtypes. Myopic maculopathy was present in 28 (43%) type 1 and five (42%) type 2 patients. The odds for higher category myopic maculopathy increased by a factor of 2.15 with each mm of axial elongation (95% CI = 1.14 to 4.04, *p* = 0.02) but not with age (odds ratio = 1.02 per year, 95% CI = 0.97 to 1.09, *p* = 0.39) in type 1.

**Conclusion:**

We find myopia in our cohort is nonprogressive. We find no difference in axial length or refractive error between subtypes. Myopic maculopathy is common, its severity depending on axial length, not age. These findings are relevant for risk stratification of vision‐threatening myopia.

## 1 INTRODUCTION

Strickler syndrome, first described by Gunnar Stickler in a family with progressive myopia, retinal detachment (RD), early onset arthropathy, hypermobility, vertebral abnormalities, and hearing loss, is a hereditary connective tissue disease. It is also associated with other findings such as retrognathia, cleft palate, flat facial profile, cataract, and glaucoma (Stickler et al., 1965; Stickler & Pugh, 1967). Large intra‐ and interfamilial clinical variability is well‐known (Ahmad et al., 1996; Zlotogora et al., 1992). The most common disease mechanism leading to Stickler syndrome is haploinsufficiency of type II collagen, caused by monoallelic pathogenic variants in the *COL2A1* gene inhibiting normal alpha‐1(II)procollagen homotrimer formation (Francomano et al., 1987; Richards et al., 2006). Collagen type II is present in cartilage, the vitreous body, the inner ear, the nucleus pulposus, and embryonal skeletal ossification (He et al., 2019). The lack of collagen type II in the vitreous leads to the appearance of an empty vitreous with visible membranes (Richards et al., 2010; Snead & Yates, 1999). Stickler syndrome caused by variants in *COL2A1* is called type 1 (STL1). Less frequently Stickler syndrome presents with a different kind of empty vitreous, the beaded type, named type 2 Stickler syndrome (STL2). STL2 is linked to pathogenic variants in the *COL11A1* gene coding for alpha‐1(XI)procollagen, part of the type XI collagen heterotrimer (Richards et al., 1996; Snead et al., 1994). Autosomal recessive types of Stickler syndrome have been described on a case basis with disease‐causing variants in the genes coding for collagen type IX (*COL9A1*: STL4; *COL9A2*: STL5; *COL9A3*: STL6) (Baker et al., 2011; Faletra et al., 2014; Van Camp et al., 2006).

Optically empty vitreous is seen in all patients with Stickler syndrome even though it can be difficult to diagnose after posterior vitreous detachment. Myopia is thought to be the second most prominent feature, affecting up to 80% of patients, the majority having a refractive error of ≤−6.00 Dioptres (D) (Boysen et al., 2020; Parma et al., 2002). Myopia is defined as a condition where the eye's refractive power is too strong, leading to image formation in front of the retina, and can be due to increased axial length (AL) of the eye above 22–24 mm. High myopia is equal to a spherical equivalent refractive error (SER) of ≤−6.00 D. High myopia due to axial elongation is a risk factor for pathologic myopia including myopic maculopathy, posterior staphylomas, and optic neuropathy (Atchison et al., 2004; Flitcroft et al., 2019). Myopic maculopathy is a vision‐threatening condition and is classified into five categories based on fundus photographs or funduscopy (Ohno‐Matsui et al., 2015).

Myopia is a well‐known feature of Stickler syndrome, but several important aspects remain unknown. There is still confusion on whether myopia is progressive, and we lack knowledge on the association between AL, refractive error, and complications to myopia in Stickler syndrome. This article will explore the prevalence and degree of myopia, if myopia is progressive, and look at myopic maculopathy as a possible complication of myopia in patients with genetically confirmed STL1 and STL2.

## 2 METHODS

### 2.1 Data collection

We present data from a cross‐sectional study on 84 patients with Stickler syndrome performed at the Department of Ophthalmology at Copenhagen University Hospital – Rigshospitalet, Denmark. The cross‐sectional examination was combined with historical data from medical files. Patients were recruited from all of Denmark. Recruitment, data collection, and examination occurred between 1 May 2023, and 30 September 2024. The primary outcomes of the study were AL, refractive error (reported as SER = spherical refraction + ½ × cylindrical refraction), SER over time, and myopic maculopathy grade in Danish patients with genetically confirmed STL1 or STL2.

### 2.2 Participants

We included patients with clinically diagnosed Stickler syndrome, genetically confirmed either by previous genetic workup or consent to undergo genetic testing as part of this study. Denmark does not have a national database or diagnostic code specifically for Stickler syndrome, which makes it necessary to hand‐search eligible patients. To minimize selection bias towards patients with Stickler syndrome with severe eye disease, we recruited participants from Ophthalmology Departments, Departments of Genetics, Departments of Ear Nose Throat, the Centres for Lip and Palate Clefts, and the Centres for Rare Diseases from all of Denmark. Relevant databases (Danish Family Archive for Genetic Eye Disease, RAREDIS® – the Nordic database for rare and genetic diseases, and genetic databases) were screened. We advertised in patient organizations for Stickler syndrome, patient organizations for visual impairment, the Network for Public Optometrists, and the Danish Facebook group for Stickler syndrome ‘Stickler Syndrom – os med sygdommen inde på livet’.

### 2.3 Genetic analysis

Variants from prior obtained genetic test results were re‐evaluated based on the American College of Medical Genetics and Genomics and the Association for Molecular Pathology (ACMG) guidelines (Richards et al., 2015). In patient cases without a certain genetic diagnosis, a targeted next‐generation sequencing (NGS) whole genome or exome‐based panel on Stickler syndrome‐related genes was performed using virtual gene panels for the following genes: *COL11A1, COL11A2, COL18A1, COL2A1, COL9A1, COL9A2, COL9A3, COMP, ATOH7, BEST1, BMP4, CAPN5, CTC1, CTNNB1, FZD4, GZF1, KCNJ13, KIF11, LOXL3, LRP2, LRP5, MATN3, NDP, NR2E3, P3H2, RS1, SLC26A2, TSPAN12, VACN*, and *ZNF408*. In families with a known genetic cause all at‐risk persons were offered testing for the specific variant.

### 2.4 Ophthalmic examination

After anterior segment slit‐lamp examination and Goldmann applanation tonometry, eyes were dilated (Adults: 1% Tropicamide and 10% phenylephrine hydrochloride; children under 18: 0.5% Tropicamide and 2.5% phenylephrine hydrochloride). Ocular biometry using IOLMaster 700 (Carl ZEISS Meditec, Jena, Germany) was performed, ultra‐wide‐field colour and blue fundus photographs (Clarus 500, Carl ZEISS Meditec USA Inc., CA, USA), and posterior segment slit‐lamp examination including fundoscopy (Digital Wide Field Lens, VOLK, Mentor, OH, USA) followed. Two examiners (KB and LK) graded the fundus photographs for myopic maculopathy according to the META‐PM Study Group International Photographic Classification and Grading System for Myopic Maculopathy (Category 0: no myopic retinal degenerative lesion. Category 1: tessellated fundus. Category 2: diffuse chorioretinal atrophy. Category 3: patchy chorioretinal atrophy. Category 4: macular atrophy. ‘Plus’ lesions: Lacquer cracks, choroidal neovascularization, and Fuchs spots) (Ohno‐Matsui et al., 2015). Fundus photographs were gradable only when they showed the entire optic nerve and macula with visible details. Cases with interobserver grading differences were discussed until agreement was reached. Staphylomas were diagnosed on high‐speed infrared dense posterior pole scans (ART 11 frames and 61 sections, 30° × 25°, with and without EDI mode, Heidelberg SPECTRALIS, Heidelberg Engineering, Heidelberg, Germany). Pre‐ and post‐dilation refraction was measured using a handheld Autorefractor (Retinomax K‐plus 3, Righton Ophthalmic Instruments, Tokyo, Japan).

### 2.5 Medical record review

Patients younger than 4 years did not undergo ophthalmologic examinations, but when available, results were extracted from medical records (e.g., SER, AL measurements, fundus photographs). Patients who decided against eye examination could contribute with medical records alone. SER over time was retrieved from medical records with one measurement for every calendar year. If several SER were available for the same calendar year, the most objective SER was chosen in the following order: cycloplegic autorefraction or retinoscopy, noncycloplegic objective refraction, subjective refraction, and spectacle or contact lens correction. SER over time was cropped after cataract surgery, refractive surgery, or RD repair with scleral buckle.

### 2.6 Statistics

Statistical calculations were made using RStudio Version 1.4.1717. Statistical significance levels were set at *p* < 0.05. Continuous variables were presented with mean and standard deviation (SD), or median values and interquartile ranges (IQRs), depending on normal distribution. Whenever available, right eye data were used. Left eye AL was used in case of prosthesis or scleral buckle in the right eye. For SER evaluation, we used the latest available, treatment‐naïve SER in the right eye (cropped after refractive, cataract, or RD surgery). For SER over time, we used left eye data when right eye data were cropped or missing due to missing information or prior surgery. There was no statistically significant difference between the right and left eye for AL or SER in treatment‐naïve eyes (paired *t*‐test and Wilcoxon signed‐rank test, respectively). Many patients were from larger families with Stickler syndrome and shared the same underlying genetic variant. As we still lack knowledge of phenotype–genotype correlation we decided to treat data nested by disease‐causing variants, using nested mixed‐effect model or cumulative link mixed model for regression analyses on data with continuous or ordinal outcome variables respectively.

### 2.7 Ethics statement

The study was conducted following the Declaration of Helsinki and approved by the Capital Region Danish Research Ethics Committees (H‐21033000). Per Article 30 GDPR, the scientific project is registered in the Capital Region of Denmark's record of processing activities (P‐2022‐152). All patients provided written consent. Patients younger than 18 years gave verbal consent to participate and written consent was obtained from their parents or legal guardians.

## 3 RESULTS

We included 84 patients (STL1 = 71, 42% male, STL2 = 13, 69% male). Median age for STL1 patients was 29 (IQR = 15–49) years, and 27 (IQR = 9–33) years for STL2. The patients came from 34 families (family size = 1–8 members) with STL1 and six families (family size = 1–4 members) with STL2. Each family had individual disease‐causing variants. Eight STL1 patients contributed with medical records alone, hereof four due to age under 4 years.

### 3.1 Refraction

**FIGURE 1:**
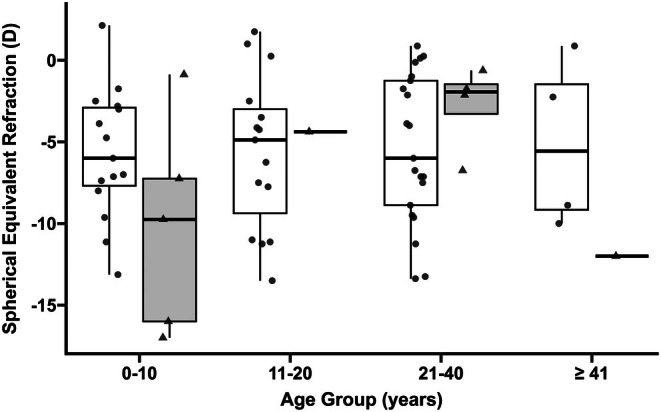
**Spherical equivalent refraction in Stickler syndrome.** Combines box and jitter plot showing the spherical equivalent refraction (SER) in D (dioptres) for different age groups in Stickler syndrome type 1 (white) and 2 (light grey).

For STL1, we found a median SER for right eyes of −6.00 (IQR = −8.88 to −2.19) D in 55 patients (age in years: median = 17, IQR = 8.5–31). For STL2, we found a median SER for right eyes of −6.75. (IQR = −10.88 to −1.94) D in 11 patients (age in years: median = 14, IQR = 7 to 29), Figure 1. There was no difference between right and left eye SER (STL1: *p* = 0.44, STL2: *p* = 0.84, Wilcoxon signed‐rank test). There was no difference between STL1 and STL2 right eye SER (*p* = 0.52, Wilcoxon rank sum test).

**FIGURE 2:**
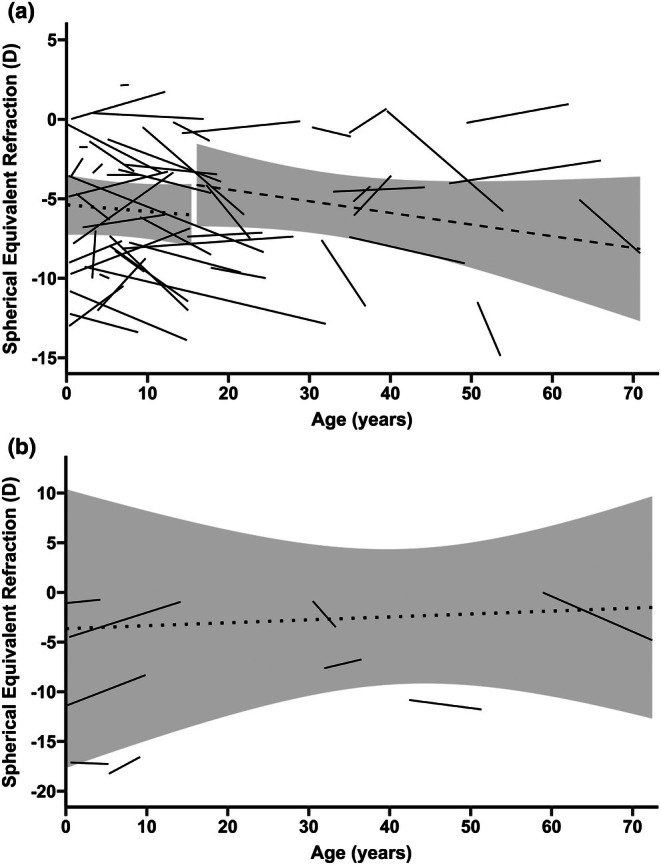
(a) **Spherical equivalent refraction over time for Stickler syndrome type 1.** Spaghetti plot on spherical equivalent refraction (SER) over time for Stickler syndrome type 1 with fitted linear regression model for each patient. Dotted line with 95% confidence interval (CI) shows the group level estimated SER change over time found by applying a mixed‐effect model (nested by disease‐causing variant and patients) to all SER measurements obtained in patients ≤15 years of age. Dashed line with 95% CI shows the group level estimated SER change over time found by applying a mixed‐effect model (not nested due to few measurements) to all SER measurements obtained in patients >15 years of age. (b) **Spherical equivalent refraction over time for Stickler syndrome type 2.** Spaghetti plot on spherical equivalent refraction (SER) over time for Stickler syndrome type 2 with fitted linear regression model for each patient. Dotted line with 95% confidence interval (CI) shows the group level estimated SER change over time found by applying a mixed‐effect model (nested by disease‐causing variant and patients) to all SER measurements obtained in patients with STL2.

Medical record review provided at least two treatment‐naïve SER values for 50 patients with STL1 (in 11 patients we used left eye data). The same was true for nine STL2 patients (three with left eye data). The median retrospective follow‐up time for SER measurements was 9 (IQR = 0.5–30) years in STL1 and 4.5 (IQR = 1–14) years in STL2. SER over time for STL1 and STL2 is presented in Figure 2. When applying a mixed effects model, nested by disease‐causing variant and patients, on repeating SER measurements taken before the age of 16 years on STL1 patients (31 patients, 4 left eyes), we found no statistically significant change in SER with age (estimate of annual change in D = −0.04, SE = 0.06, *p* = 0.49). For patients with STL2, we only had nine patients with repeated measurements in treatment‐naïve eyes. When applying a mixed effects model, nested by disease‐causing variant and patients, we found the change of SER with time was not statistically significant either (estimate of annual change in D = 0.03, SE = 0.15, *p* = 0.84).

### 3.2 Axial length

**FIGURE 3:**
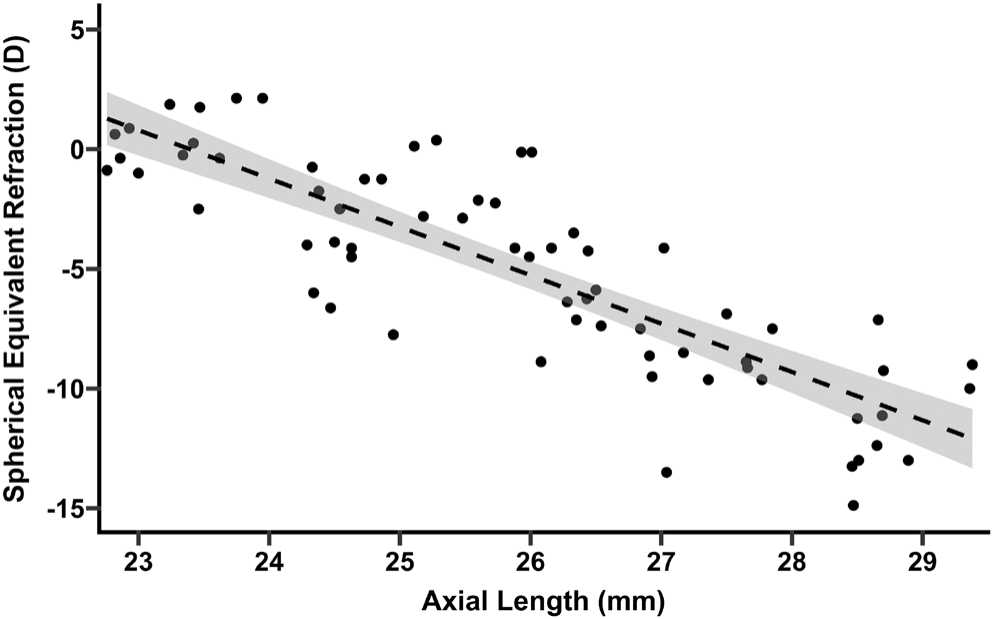
**Spherical equivalent refraction and axial length in Stickler syndrome type 1.** Relationship between spherical equivalent refraction (SER) and axial length (AL) in Stickler syndrome type 1 with linear regression model (dashed line with standard error).

In the cross‐sectional examination, AL measurements were available in at least one eye of 61 STL1 patients (age in years: median = 32, IQR = 15–49), and 12 STL2 patients (age in years: median = 28, IQR = 9–33.75), excluding eyes with scleral buckle. For STL1 there was no difference in AL between right eyes (mean = 25.93 ± 1.96 mm) and left eyes (mean = 25.93 ± 2.01 mm; *p* = 0.31, paired *t*‐test). The same was true for STL2 right (mean = 26.55 ± 3.45 mm) and left (mean = 26.58 ± 3.48 mm) eyes (*p* = 0.32). Thus, AL measurements for right eyes with scleral buckle or missing measurements were replaced with treatment‐naïve AL of the left eye when available. The mean AL for STL1 was 25.97 ± 1.98 mm (95% CI = 25.46–26.47 mm) and 26.55 ± 3.45 mm for STL2 (95% CI = 24.36–28.74 mm). We found no difference in AL between Stickler syndrome subtypes (*p* = 0.58, Welch two sample *t*‐test). For STL1 a 1 mm increase in AL equalled a −2.02 D change in SER (SE = 0.15 D, 95% CI = −2.33 to −1.71 D, *p* < 0.001), Figure 3.

### 3.3 Myopic maculopathy

**FIGURE 4:**
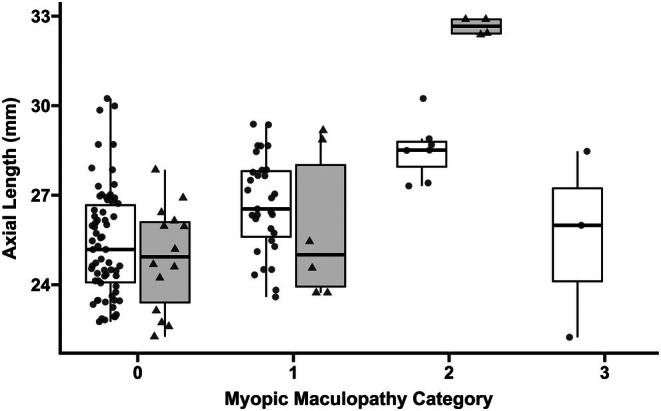
**Axial length by myopic maculopathy category in Stickler syndrome types 1 and 2.** Combined jitter and boxplot showing the axial length (mm) for different categories of myopic maculopathy in Stickler syndrome type 1 (white) and 2 (light grey). The figure includes both right and left eyes. Myopic maculopathy category: 0 = no myopic maculopathy; 1 = tessellated fundus; 2 = diffuse chorioretinal atrophy; 3 = patchy chorioretinal atrophy; 4 = macular atrophy (Ohno‐Matsui et al., 2015).

Fundus photos could be taken in at least one eye of 65 patients with STL1 (six patients could not cooperate: four due to low age, one due to low visual acuity, and one who only wanted to participate with medical records) and 12 with STL2 (one could not cooperate). Their median age was 31 (IQR = 17–49) years for STL1 and 28 (IQR = 10–34) years for STL2. The results of myopic maculopathy grading are presented in Table 1, showing that 37 (57%) of STL1 and seven (58%) of STL2 patients had no myopic maculopathy in either eye (Category 0). The odds ratio of having a higher category of myopic maculopathy in STL1 was 2.15 (95% CI = 1.14–4.04, *p* = 0.02) for each 1 mm increase in AL including both right and left eyes (cumulative link mixed model, nested for disease‐causing variant and patient). However, age did not influence the myopic maculopathy category (odds ratio = 1.03 per year, 95% CI = 0.97–1.09, *p* = 0.35). This model could not be applied to STL2 due to the small sample size. Figure 4 shows AL for each myopic maculopathy category in both subtypes. Regardless of the Stickler syndrome subtype, no patient showed any ‘plus’ lesions (lacquer cracks, choroidal neovascularization, or Fuchs spot) or had a macula hole or foveoschisis.

**TABLE 1:** Myopic maculopathy in type 1 and type 2 Stickler syndrome.

Category^a^	STL1					STL2				
	0	1	2	3	Total no.	0	1	2	3	Total no.
Patients^b^	37 (57%)	19 (29%)	5 (8%)	4 (6%)	65	7 (58%)	3 (25%)	2 (17%)	0	12
Right eye^c^	35 (61%)	16 (28%)	5 (9%)	1 (2%)	57	7 (58%)	3 (25%)	2 (17%)	0	12
Left eye^d^	35 (59%)	17 (29%)	3 (5%)	4 (7%)	59	7 (58%)	3 (25%)	2 (17%)	0	12
RD	22 (30%)	7 (21%)	2 (25%)	1 (20%)	32	0 (0%)	3 (50%)	0 (0%)	0	3

*Note*: The table represents the distribution of myopic maculopathy categories in Stickler syndrome types 1 and 2, as well as the prevalence of previous RD for each category.

Abbreviations: RD, Retinal detachment; STL1, Stickler syndrome type 1; STL2, Stickler syndrome type 2.

^a^ Myopic maculopathy category after the proposed system by the META‐PM study group (Ohno‐Matsui et al., 2015). Category 0 = no myopic maculopathy; 1 = tessellated fundus; 2 = diffuse chorioretinal atrophy; 3 = patchy chorioretinal atrophy; 4 = macular atrophy.

^b^ Reporting the highest myopic maculopathy category for the right and left eye in a patient.

^c^ STL1: 8 eyes missing (no cooperation: 1, prosthesis: 4, unclear media: 3).

^d^ STL1: 6 eyes missing (prosthesis: 1, unclear media: 5).

Staphylomas were present in three patients with STL1 (Patient 1: Staphyloma in both eyes, AL = 29.36 mm and 29.38 mm. Patient 2: Staphyloma in the left eye, AL = 26.91 mm. Patient 3: Staphyloma in the left eye, AL = 27.91 mm) and two patients with STL2 (Patient 1: Staphyloma in both eyes, AL = 26.42 mm and 25.95 mm. Patient 2: Staphyloma in the right eye, AL = 25.19 mm).

Epiretinal membrane or fibrosis was present in 22 eyes (20%) of STL1 (five patients had bilateral changes). OCT was unattainable in 32 eyes, in 16 due to noncompliance, and in 16 due to technical difficulties (prosthesis, extensive retinal changes, or poor vision). The median age for STL1 patients with epiretinal fibrosis in at least one eye was 39 (IQR = 29–50) years and their mean AL was 25.86 ± 2.00 mm. Previous RD was seen in 13 (59%) eyes with epiretinal fibrosis. For eyes with epiretinal fibrosis, there was no difference in AL when considering previous RD (mean AL for RD = 25.77 mm, mean AL for non‐RD = 25.99 mm, *p* = 0.80, *t*‐test). No patient with STL2 had epiretinal fibrosis.

## 4 DISCUSSION

This cross‐sectional study combined an updated eye examination with retrospective data from medical record reviews of 84 Danish patients with genetically confirmed types 1 and 2 Stickler syndrome to evaluate myopia development and the association between myopia degree and myopic complications.

While myopia prevalence is rising in many parts of the world, we do not find this development in Denmark (Hansen et al., 2021). Furthermore, there is increasing focus on progressive myopia. To halt progressive myopic development and the associated ocular risks, including potentially vision‐threatening conditions, several methods are being researched (Morgan et al., 2012). There is limited data in the literature as to the progressive or nonprogressive nature of myopia in Stickler syndrome. A study from 1996 did not report any progression in refractive errors in 17 children with clinically diagnosed Stickler syndrome based on the presence of cleft palates (Wilson et al., 1996). The study was limited by short follow‐up during early childhood, no knowledge of possible progression in adolescence, and missing genetic verification of the Stickler syndrome diagnosis and subtype. Our study on patients with genetically confirmed Stickler syndrome did not show an overall change in refractive error with age. Collagen II is the major collagen in the vitreous body but also plays a role in the sclera during embryogenesis. Collagen XI plays an important role in regulating fibril diameter and assembly of collagen II (Luo et al., 2019). We hypothesize that changes in collagens II and XI play a strong role in the embryonic development of the eye, rather than on the growth of the eye in childhood, potentially explaining why myopia in Stickler syndrome would be nonprogressive. We found a mean AL of 25.96 mm for STL1 and 26.55 mm for STL2. When looking closer into AL of the right eye according to age in STL1 we find that children under 5 years of age (two patients) have a median AL of 25.58 mm (IQR = 25.38–25.78 mm), 5–10‐year‐olds (six patients) have a median AL of 25.52 mm (IQR = 24.38–27.46 mm), 11–16‐year‐olds (10 patients) have a median AL of 26.16 mm (IQR = 23.94–27.65 mm), and participants over the age of 16 years (49 patients) had a median AL of 25.73 mm (IQR = 24.32–27.39 mm). When applying Kruskal–Wallis we find no statistically significant evidence that there is a difference in AL between the age groups (*p* = 0.99). In comparison, normative data from three large European epidemiological cohorts found a mean AL of 22.36 ± 0.75 mm for 6‐year‐olds, 23.10 ± 0.84 mm for 9‐year‐olds, 23.41 ± 0.86 mm in 15‐year‐olds, and 23.67 ± 1.26 in adults (Tideman et al., 2018). Our data indicate that AL in patients with Stickler syndrome is stable across all ages and does not show the normal growth seen in the general population during childhood, but the ALs in children with Stickler syndrome are higher than normal. High myopia (≤−6.00 D, ≥26 mm) in children under 10 years is associated with ocular and systemic disorders, so‐called secondary high myopia, such as Stickler syndrome (Wang et al., 2020). The present study had retrospective SER data on 62 patients with STL1 before the age of 10 years. When looking at their median SER before the age of 10 years we found a median SER of −5.91 (IQR = −8.50 to −2.91) D. For STL2 (six patients) the median SER was −9.26 (IQR = −15.19 to −3.83) D. High myopia is also associated with an increased risk of vision loss due to myopic maculopathy, posterior staphyloma, myopic traction maculopathy, optic neuropathy, and RD (Flitcroft et al., 2023). The Copenhagen City Eye Study found myopic maculopathy to be a major cause of visual impairment and blindness in adults before the age of 65 years (Buch et al., 2004). Myopic maculopathy category ≥2 is considered pathologic myopia, which this study found in at least one eye of 14% of STL1 and 17% of STL2 patients. A German study by Hopf et al. from 2020 found a prevalence of myopic maculopathy (category ≥2) of 9.4% among 519 adults with a SER ≤−6.00 D using the META‐PM classification, but only 0.5% in the control group (authors calculation based on data available in supplemental material eTable 2). Furthermore, we found a 2.15 odds ratio for higher myopic maculopathy category per one 1 mm increase in AL, comparable to a recent Dutch study on 626 adults with nonsystemic high myopia, that found an odds ratio of 2.35 (Haarman et al., 2022). On the other hand, our study did not find a statistically significant risk for higher category myopic maculopathy with increasing age in contrast to several large studies investigating the risk factors for myopic maculopathy in patients without Stickler syndrome (Fang et al., 2018; Hayashi et al., 2010; Hopf et al., 2020; Mu et al., 2025; Ohno‐Matsui et al., 2021; Xiao et al., 2018). This might be because myopia in Stickler syndrome is congenital and nonprogressive, indicating that AL has more influence on myopic maculopathy degree than age. However, direct comparison is not possible, as studies on non‐Stickler populations predominantly include adult patients with high myopia (≤−6.00 D and/or ≥26 mm AL). A prospective study on patients with Stickler syndrome is needed to validate the tendencies we found. Staphylomas were less common than expected based on common knowledge on staphylomas in myopic eyes in general, with only three STL1 and two STL2 patients affected (AL varied between 25.19 and 29.38 mm). This is comparable to the results of a French study from 2021, which found posterior staphyloma in 11.5% of patients with Stickler syndrome and high myopia (AL ≥26 mm) compared with 68.0% in a non‐Stickler control group matched by AL (Xerri et al., 2021). No patient had a CNV, macular hole, or posterior retinoschisis. Myopic traction maculopathy includes lamellar holes, macular foveoschisis, and epiretinal fibrosis. We found epiretinal fibrosis in 20% of STL1 eyes, of which 59% had previous RD. No epiretinal fibrosis was observed in STL2 patients. Lamellar holes and foveoschisis possibly happen due to strong attachments of the inner retina to the vitreous cortex (Ohno‐Matsui et al., 2021). We did not observe these complications in our patients, which might be due to the altered vitreous body in Stickler syndrome. There is a well‐established association between SER and AL, and as a rule each 1 mm increase in AL corresponds to three dioptres decrease in refractive power (Atchison et al., 2004). However, in our STL1 patients, we found that a 1 mm increase in AL corresponded to a 2.02 D decrease in refractive power. In other words, we found STL1 patients with longer AL than expected based on their refractive error alone. Regarding this, we would postulate that patients with Stickler syndrome can have a longer AL than expected. This further underlines the importance of AL measurement and further anterior segment studies would be needed to show a correlation. A limitation to our study was the retrospective approach to evaluate myopia progression; ideally prospectively collected data for AL and myopia‐related complications should be used. We used treatment‐naïve SER to evaluate change in refractive error with age (data were cropped after refractive surgery, cataract surgery, RD surgery, and enucleation). Most participants' follow‐up ended before the age of 30 years, which reflects the time that these surgeries take place in patients with Stickler syndrome. This resulted in only a few data on possible progressions in SER during adulthood. Myopia progression usually takes place in childhood and adolescence, and progression in adults is mostly linked to environmental factors in the background population (Bullimore et al., 2023; Flitcroft et al., 2019; Hansen et al., 2021; Hardy et al., 2013). Hence, we consider this only a minor limitation with no influence on our conclusions. Another limitation is the sample size for STL2 that makes statistical calculations difficult. This should be kept in mind when drawing conclusions. Lastly, we used a multi‐source recruitment process to avoid bias towards predominantly including patients with severe eye disease and high myopia, which might have occurred when only recruiting through ophthalmological departments. This effort is reflected in our results with lower median SER than in previous studies on Stickler syndrome that found mean refractive errors between −8.70 to −14.50 D (Kondo et al., 2016, Nixon et al., 2025, Wang et al., 2016, Wubben et al., 2018, Zhou et al., 2018). Still, we do not know if nonresponders to the study invitation were more, or less affected by their disease than participants. High myopia and maculopathy could influence accessibility to participate in research projects. On the other hand, mildly affected individuals might not see a need for their participation or even know of their disease.

This retrospective and cross‐sectional study on genetically confirmed patients with Stickler syndrome indicates that myopia in Stickler syndrome does not progress with age in general, that 14–17% of patients have pathologic myopic maculopathy, and that the degree of myopic maculopathy depended on AL but not age in STL1. The mean AL of Stickler syndrome patients was above normal and greater than expected based on refraction alone, regardless of the Stickler syndrome subtype. Thus, AL measurements are invaluable in all patients with Stickler syndrome.

## FUNDING INFORMATION

This research was supported by ‘Fight for Sight’ (Øjenforeningen), ‘Synoptik Fonden’, ‘Candys Foundation’, ‘Helsefonden’, ‘Jascha Fonden’, ‘Wholesaler Christian Andersen and wife Ingeborg Ovidia Signe Andersen, born Schmidts Grant’, ‘Helene and Viggo Bruuns Foundation’, and ‘Master Carpenter Jørgen Holm and wife Elisa, born Hansen Memorial Grant’. The funders had no role in the design and conduct of the study; collection, management, analysis, and interpretation of the data; preparation, review, or approval of the manuscript; and decision to submit the manuscript for publication.

## GUIDELINES

This manuscript follows the STROBE reporting checklist for cross‐sectional studies.

## Supporting information


Appendix S1
